# Guanylate-binding proteins balance iNOS/Arg-1 in myeloid cells during *L. major* infection and promote host defense to infection

**DOI:** 10.1128/mbio.02825-25

**Published:** 2026-03-10

**Authors:** Lucy Fry, Het Adhvaryu, Hayden Roys, Anne Bowlin, Gopinath Venugopal, Jordan T. Bird, Charity Washam, Masahiro Yamamoto, Jörn Coers, Stephanie Byrum, Daniel Voth, Tiffany Weinkopff

**Affiliations:** 1 Department of Microbiology and Immunology, College of Medicine, University of Arkansas for Medical Sciences 155638, Little Rock, Arkansas, USA; 2 Department of Biochemistry and Molecular Biology, College of Medicine, University of Arkansas for Medical Sciences 155638, Little Rock, Arkansas, USA; 3 Arkansas Children's Research Institute 366944 , Little Rock, Arkansas, USA; 4 Department of Immunoparasitology, Research Institute for Microbial Diseases, Osaka University 34822, Suita, Osaka, Japan; 5 Department of Molecular Genetics and Microbiology, Duke University Medical Center 609772, Durham, North Carolina, USA; 6 Department of Integrative Immunobiology, Duke University Medical Center 609772, Durham, North Carolina, USA; University of Wisconsin-Madison, Madison, Wisconsin, USA

**Keywords:** leishmaniasis, *Leishmania*, guanylate-binding proteins, macrophages

## Abstract

Cutaneous leishmaniasis (CL) is a debilitating neglected tropical disease characterized by lesions that can range from self-healing to permanent disfiguration. A predominant Th1 response, which stimulates IFN-γ production, is crucial for parasite control during self-healing CL. While IFN-γ primarily activates macrophages to produce nitric oxide via inducible nitric oxide synthase (iNOS), leading to parasite control, it also activates other downstream pathways involved in cell autonomous immunity. One such pathway is the activation of guanylate-binding proteins (Gbps), a class of interferon-inducible GTPases. However, the role of Gbps during CL has been minimally explored. Utilizing RNA-Seq, we found that *Leishmania major* infection leads to the upregulation of several Gbps in C57BL/6 mice. *In vitro* studies using Gbp^Chr3^ knockout (KO) and C57BL/6 control mice reveal that bone marrow-derived macrophages from KO mice exhibit higher parasite burdens following IFN-γ treatment, independent of Gbp localization to the parasite. Single-cell RNA-Seq identifies macrophages as the primary expressers of Gbps during *L. major* infection *in vivo. In vivo*, Gbp^Chr3^ KO mice display increased disease severity and parasite load. Gbp^Chr3^ KO macrophages and monocytes show elevated ARG-1 and reduced iNOS expression, indicating a shift toward a parasite-permissive environment that supports parasite growth. These findings highlight a critical role for Gbps in immune-mediated control of CL.

**IMPORTANCE:**

*Leishmania* parasites cause cutaneous lesions that are often resistant to drug treatment, and no vaccine is currently available, highlighting the need to better understand host mechanisms that control infection. In this manuscript, we explore the role of guanylate-binding proteins (Gbps) in host macrophages during *Leishmania major* infection. We demonstrate that Gbps are critical for host defense both *in vitro* and *in vivo*. Notably, this protection is independent of Gbp localization to the parasite, revealing a novel aspect of Gbp biology. Instead, we find that differences in parasite burden and disease severity in Gbp-deficient mice are linked to altered activation of tissue macrophages and monocytes. Our findings suggest that Gbps coordinate inducible nitric oxide synthase expression in macrophages, the primary cells that house and control *Leishmania* parasites, and play a unique immunoregulatory role during infection.

## INTRODUCTION

Cutaneous leishmaniasis (CL) represents the spectrum of diseases caused by vector-transmitted *Leishmania* protozoan parasites endemic to the tropical and subtropical regions of the world (1). Each year, there are 1–2 million new cases of CL with 12 million ongoing infections in areas, including Central and South America, the Mediterranean basin, the Middle East, and Central Asia (2, 3). The species of parasite and the host immune response dictate clinical disease within the spectrum of CL. Both overactive and insufficient immune responses can lead to chronic disease (4). Specifically, control of the intracellular infection is dependent on an effective CD4^+^ Th1 immune response (5). Inability to initiate a dominant Th1 response is a major cause of chronic non-healing disease, where mice developing a Th2 response are more susceptible to *Leishmania* parasites (6).

During infection with *Leishmania major* parasites, a strong Th1 immune response characterized by high levels of IFN-γ is associated with better parasite control (7–9). IFN-γ is necessary for the control of *Leishmania* infection, as mice lacking the IFN-γ receptor are unable to control *L. major* infection (7). Additionally, mice lacking IFN-γ are more susceptible to chronic *Leishmania amazonensis* infection compared to control mice (9). Interestingly, during *L. major* infection, the requirement for IFN-γ signaling is mainly isolated to myeloid lineage cells (8). For instance, mice selectively lacking the IFN-γ receptor on macrophages result in uncontrolled lesion growth following infection with *L. major* (8). This is most likely because IFN-γ stimulates macrophages, the main cell type harboring *Leishmania* parasites, to produce effector molecules like nitric oxide (NO) and reactive oxygen species (ROS), which directly limit parasite growth (10–12). In addition, IFN-γ activates a number of other cell-autonomous programs, many of which have not been investigated during CL. For instance, IFN-γ stimulates the expression of guanylate-binding proteins (Gbps) (13). Human GBPs and murine Gbps are a part of the IFN-inducible dynamin family of GTPases that exert multifactorial roles during disease and infection (13). These proteins are characterized by their ability to bind and hydrolyze GTP, but Gbps also play other significant roles in cells, such as disrupting pathogen membranes, inducing inflammasome activation, and promoting pathogen cell death (13, 14). Specifically, Gbps possess pattern recognition capabilities and exert antimicrobial effects through their GTPase activity against bacteria, viruses, and parasites (15–19).

Gbps localize to both host cell membranes and target intracellular pathogen membranes during infections, such as *Toxoplasma gondii* and *Shigella flexneri* infection, leading to membrane disruption, exposure of the pathogen to the intracellular environment, or pathogen death (15–21). Ultimately, membrane disruption leads to the recruitment of supramolecular structures to activate cell death pathways, such as apoptosis or pyroptosis, through inflammasome formation and release of pro-inflammatory molecules (17). Gbps also aid in the recruitment of antimicrobial effectors to pathogen-containing vacuoles, like NADPH oxidase subunits, and they facilitate the capture of intracellular microbes in autophagolysosomes (22–24). Additionally, Gbps can inhibit viral replication and suppress proteolytic activity of furin, a proprotein convertase that cleaves viral pro-proteins, further disrupting viral maturation (25, 26). Importantly, Gbps have been proposed as a new class of pattern recognition receptors binding directly to LPS; however, additional ligands recognized by Gbps are still largely unknown (19, 27–29). Gbps also exert anti-microbial effects without localizing to the pathogen membrane (30, 31). For example, during *Chlamydia muridarum* infection, Gbps do not localize to the vacuole, but they are still required for rapid induction of pyroptosis in response to *Chlamydia* infection (30).

To date, minimal work has been done to understand the role of murine Gbps during *Leishmania* infection. *Gbp2* and *Gbp5* are upregulated during infection with *L. major* in susceptible non-healing female BALB/c mice (32). Additionally, during *Leishmania donovani* infection in non-phagocytic cells, IFN-γ upregulates Gbp2, which is responsible for restriction of growth, despite Gbp2 not localizing to the parasitophorous vacuole (PV) (31). Importantly, the expression of human GBP1 (hGBP1), hGBP2, hGBP4, and hGBP5 is elevated in human CL lesions (33, 34). However, the role of GBPs in cell-autonomous defense against *Leishmania* species causing CL is incompletely understood, leaving a wide knowledge gap. Here, we demonstrate that the expression of Gbps is highly upregulated during murine CL. Moreover, this work shows Gbps possess a transient role in controlling parasite burdens and disease severity in an experimental model of CL due to *L. major* infection. Ultimately, we find that Gbps orchestrate the expression of inducible nitric oxide synthase (iNOS) in macrophages while selectively downregulating arginase (Arg-1), thereby contributing to better control of *L. major* parasites. Unlike *Toxoplasma* infection, our data suggest that the function of Gbps during *L. major* infection is not dependent on localization to the pathogen, highlighting a unique feature of Gbp biology in leishmaniasis.

## MATERIALS AND METHODS

### mRNA extraction and real-time PCR

mRNA was extracted with the Qiagen RNeasy Mini Kit 250 (Qiagen). RNA was reverse-transcribed with the High-Capacity cDNA Reverse Transcription Kit (Applied Biosystems). Quantitative real-time PCR (RT-PCR) was performed using SYBR green PCR Master Mix and a QuantStudio 6 Flex Real-Time PCR System (Life Technologies). Mouse primer sequences were selected from the PrimerBank (http://pga.mgh.harvard.edu/primerbank/): Gbp2 (forward 5′-CTGCACTATGTGACGGAGCTA-3′ and reverse 5′-GAGTCCACACAAAGGTTGGAAA-3′), Gbp3 (forward 5′-GAGGCACCCATTTGTCTGGT-3′ and reverse 5′-CCGTCCTGCAAGACGATTCA-3′), Gbp5 (forward 5′-CAGACCTATTTGAACGCCAAAGA-3′ and reverse 5′-TGCCTTGATTCTATCAGCCTCT-3′), Gbp7 (forward 5′-TCCTGTGTGCCTAGTGGAAA-3′ and reverse 5′-CAAGCGGTTCATCAAGTAGGAT), and Gbp9 (forward 5′-GGTCACCGGGAATAGACTGG-3′ and reverse 5′-GGGCCACACTTGTCATAGCA-3′). Relative mRNA expression was normalized to that of the RPS11 housekeeping gene (35).

### Mice

Female C57BL/6NCr mice were purchased from the National Cancer Institute. Gbp^Chr3^ KO mice were provided by Dr. Jörn Coers from Duke University and have been previously described (36). Mice were used for experiments at 6–8 weeks of age and were housed under pathogen-free conditions at the University of Arkansas for Medical Sciences (UAMS).

### Parasites

*L. major* (WHO/MHOM/IL/80/Friedlin), *L. amazonensis* (MHOM/BR/00/LTB0016), or *Leishmania mexicana* (MNYC/BZ/62/M379) were used for *in vivo* experiments. For immunofluorescence experiments, DsRed *L. major* Friedlin was utilized. Parasites were grown *in vitro* in Schneider’s Drosophila medium (Gibco) supplemented with 20% heat-inactivated fetal bovine serum (Invitrogen), 2 mM L-glutamine (Sigma), 100 U/mL penicillin, and 100 μg/mL streptomycin (Sigma). Metacyclic promastigotes from 4- to 5-day-old parasite cultures were isolated by Ficoll density gradient centrifugation (Sigma) (37).

### Generation of bone marrow-derived macrophages

Femurs collected from mice were soaked in 70% ethanol for 2 min and then flushed with 10 mL of cDMEM to extract bone marrow cells. Bone marrow cells were counted before plating 5 × 10^6^ cells per 100 mm petri dish in 10 mL of conditioned macrophage media (cDMEM with 25% L929 cell supernatants). Cells were cultured for 7 days, refreshing media at day 3. To remove the macrophages from the Petri dish, the macrophages were washed with ice-cold phosphate-buffered saline (PBS) and gently removed with a cell scraper. The collected macrophages were counted and cultured in suspension in polypropylene tubes for parasite quantification or loaded into 24-well plates on cover slips with 1 × 10^6^ cells in 1 mL cDMEM per well for Gbp localization experiments.

### *In vitro* medium supplementation

*In vitro* macrophage cultures were infected with *L. major* at an MOI of 10 for 2 h before cells were washed two times with PBS to wash away extracellular parasites. Cells were then cultured in media alone or media supplemented with 10 ng/mL IFN-γ (Peprotech) or 100 ng/mL LPS (Sigma) + 10 ng/mL IFN-γ for 72 h for parasite quantification and 48 h for Gbp localization experiments.

### Processing of cell cultures with cytospin analysis

At 72 h post-infection (hpi), approximately 100,000 macrophages cultured in tubes were added to cytospin chambers and spun onto the slide at 1,000 revolutions per minute for 5 min using an automated cytospin (company). To quantify the number of parasites per macrophage, total uninfected and infected macrophages and total parasites were calculated manually on a brightfield upright microscope (Nikon). For each sample, 100 infected macrophages were counted. To calculate the ratio of parasites per macrophage, the total parasite number was divided by the total number of macrophages, including bystander uninfected and infected, to yield the total macrophage number. This calculation represents the infection rate in each sample.

### Immunofluorescence microscopy

At 48 hpi, samples were fixed with 4% paraformaldehyde and 0.1% Triton-X before incubation with PBS and 0.5% BSA to block nonspecific binding. Samples were incubated with rabbit anti-Gbp2 antibody (Proteintech) for 1 h followed by goat anti-rabbit AF488 secondary antibody (Life Technologies) for 1 h. DAPI was added for 5 min, and then coverslips with the samples were mounted onto slides with MOWIOL mounting media. Samples were visualized using a Ti2 Eclipse microscope with a 60× oil immersion lens objective, and images were captured with a D5-QilMc digital camera (Nikon).

### Bulk RNA-Seq: sample preparation

The bulk RNA-Seq samples were prepared, and data were acquired as a part of a previous study (38). Briefly, total RNA was isolated from the cell lysate of naive and infected ears by using Qiagen’s RNeasy plus mini kit according to the manufacturer’s instructions. The CTPR Genomics and Bioinformatics Core at the Arkansas Children’s Research Institute (ACRI) prepared sequencing libraries from RNA samples using the Illumina TruSeq Stranded mRNA Sample Preparation Kit version 2 for sequencing on the NextSeq 500 platform using Illumina reagents. The quality and quantity of input RNA were determined using the Advanced Analytical Fragment Analyzer and Qubit (Life Technologies) instruments, respectively. All samples with RNA quality number values of 8.0 or above were processed for sequencing. Sequencing libraries were prepared using the TruSeq Stranded mRNA Sample Prep Kit (Illumina). Briefly, total RNA (500 ng) was subjected to polyA selection, then chemically fragmented and converted to single-stranded cDNA using random hexamer-primed reverse transcription. The second strand was generated to create double-stranded cDNA, followed by fragment end repair and addition of a single A base on each end of the cDNA. Adapters were ligated to each fragment end to enable attachment to the sequencing flow cell. The adapters also contain unique index sequences that allow the libraries from different samples to be pooled and individually identified during downstream analysis. Library DNA was PCR amplified and enriched for fragments containing adapters at each end to create the final cDNA sequencing library. Libraries were validated on the Fragment Analyzer for fragment size and quantified using a Qubit fluorometer. Equal amounts of each library were pooled for sequencing on the NextSeq 500 platform using a high-output flow cell to generate approximately 25 million 75-base reads per sample.

### RNA-Seq analysis

Data analysis was performed for a previous study (38). Following demultiplexing, RNA reads were checked for sequencing quality using FastQC (http://www.bioinformatics.babraham.ac.uk/projects/fastqc) and MultiQC (39) (version 1.6). The raw reads were then processed according to Lexogen’s QuantSeq data analysis pipeline with slight modifications. Briefly, residual 3′ adapters, polyA read-through sequences, and low-quality (*Q* < 20) bases were trimmed using BBTools BBDuk (version 38.52) (https://sourceforge.net/projects/bbmap/). The first 12 bases were also removed per the manufacturer’s recommendation. The cleaned reads (>20 bp) were then mapped to the mouse reference genome (GRCm38/mm10/ensemble release-84.38/GCA_000001635.6) using STAR (40) (version 2.6.1a), allowing up to two mismatches depending on the alignment length (e.g., 20–29 bp, 0 mismatches; 30–50 bp, one mismatch; 50–60+ bp, two mismatches). Reads mapping to >20 locations were discarded. Gene-level counts were quantified using HTSeq (htseq-counts) (41) (version 0.9.1) (mode: intersection-nonempty).

Genes with unique Entrez IDs and a minimum of ~2 counts per million in four or more samples were selected for statistical testing. This was followed by scaling normalization using the trimmed mean of M-values method (42) to correct for compositional differences between sample libraries. Differential expression between naive and infected ears was evaluated using limma voomWithQualityWeights (43) with empirical Bayes smoothing. Genes with Benjamini and Hochberg (44) adjusted *P*-values ≤0.05 and absolute fold changes ≥1.5 were considered significant.

Gene Set Enrichment Analysis was carried out using Kyoto Encyclopedia of Genes and Genomes (KEGG) pathway databases, and for each KEGG pathway, a *P*-value was calculated using a hypergeometric test. Cutoff of both *P* < 0.05 and adjusted *P*-value/FDR value < 0.05 was applied to identify enriched KEGG pathways. DEGs that are more than 1.5-fold in *L. major*-infected ears relative to uninfected controls were used as input, with upregulated and downregulated genes considered separately. Subsequently, the heat maps were generated using these genes with complex Heatmap. All analyses and visualizations were carried out using the statistical computing environment R version 3.6.3, RStudio version 1.2.5042, and Bioconductor version 3.11.

### *In vivo* infections

**Fig 1:**
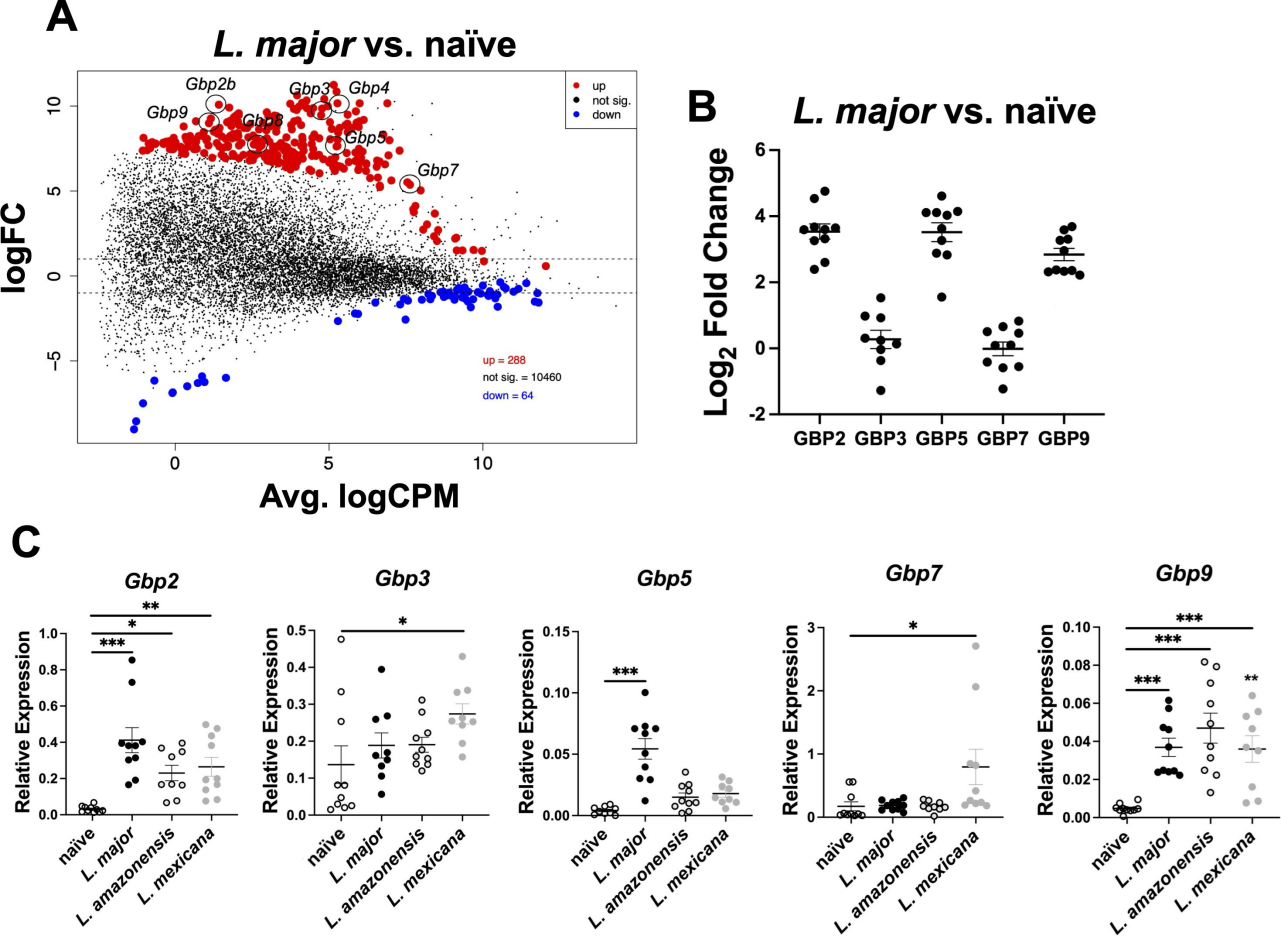
Gbp expression is a hallmark of CL. Mice were infected intradermally with 2 × 10^6^ *L. major* parasites, and at 4 weeks post-infection (wpi), RNA was isolated from ear tissue and prepared for bulk RNA-Seq. (**A**) An MDS plot depicting up- and downregulated transcripts during *L. major* infection. Red transcripts are upregulated with infection, and blue transcripts are downregulated with infection. (**B**) Real-time PCR was conducted on RNA isolated from ear lesions from naive mice or mice infected with 100,000 *L. major* parasites. Relative mRNA expression was normalized to that of the RPS11 housekeeping gene. Expression of Gbps during *L. major* infection was compared against the expression of each respective Gbp in naive mice to identify the fold change. (**C**) RT-PCR was performed on RNA isolated from ear lesions from mice infected with either 100,000 *L. major*, *L. amazonensis*, or *L. mexicana* at 6 wpi. Relative mRNA expression was normalized to that of the RPS11 housekeeping gene. Data in panels **B and C** are pooled from two independent experiments with five mice per group, and results are shown as mean ± SEM. Significance was determined using a *t*-test compared to the naive samples, where *P < 0.05 **P < 0.01, and ***P < 0.001. Significance is shown in comparison to naive samples.

**Fig 2:**
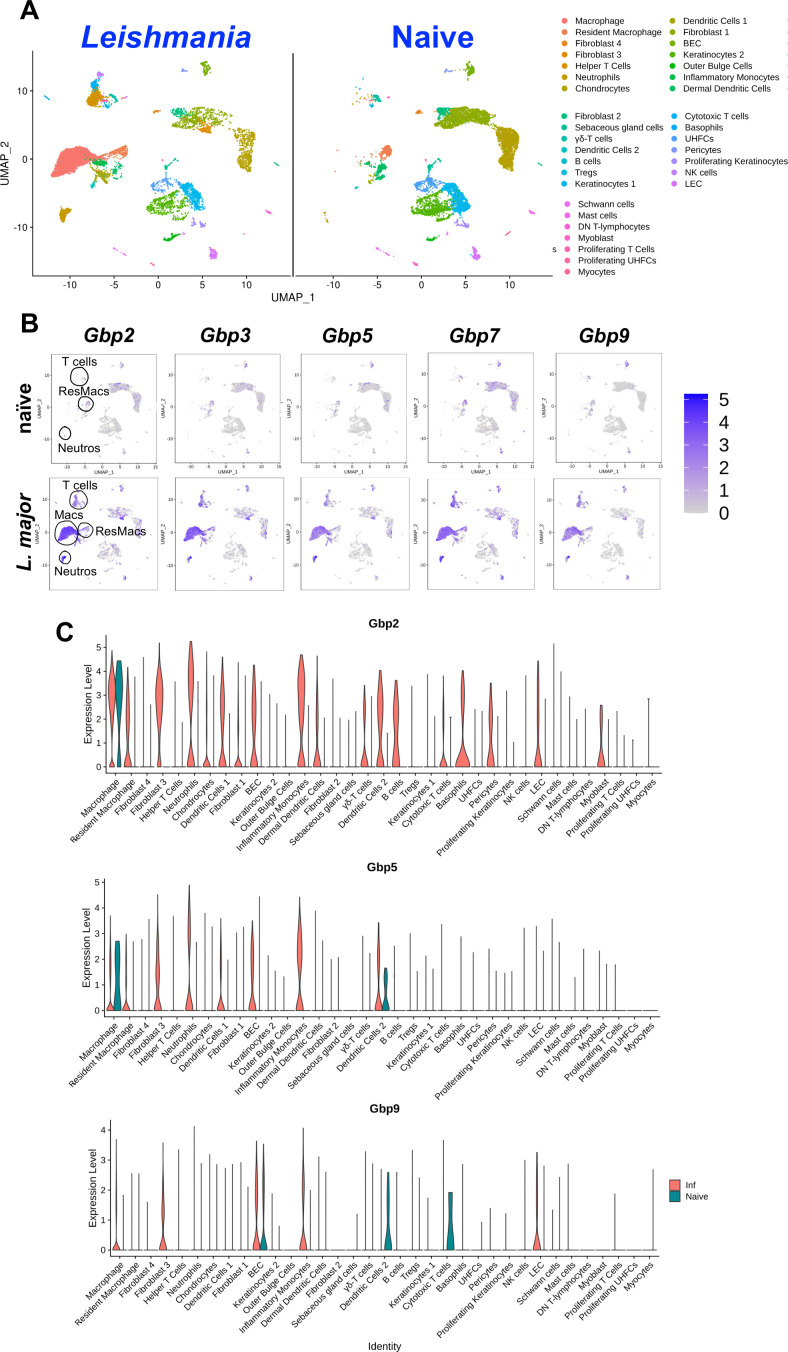
Gbp expression is associated with dermal resident macrophage and monocyte-derived macrophage populations during *L. major* infection. Mice were infected with *L. major,* and at 4 wpi, scRNA-Seq was performed on naive ear skin and CL lesions. (**A**) UMAP plots depict 35 unique cell populations that were identified in the ear dermis during *L. major* infection. (**B**) Feature plots of expression distribution for *Gbp2*, *Gbp3*, *Gbp5*, *Gbp7,* and Gbp9 in naive animals and *L. major-*infected mice. Expression levels for each gene are color-coded and overlaid onto a UMAP plot. Cells with the highest expression level are colored dark purple. (**C**) Differential expression of selected Gbp transcripts in 35 different cell types.

For infections, 100,000 (Fig. 1) or 2 × 10^6^ (all other figures) parasites were intradermally injected into the right ear in a volume of 10 μL of PBS (Gibco). The contralateral left ear was not injected with parasites, serving as an uninflamed control. Ear thickness and lesion diameters were recorded weekly with electronic calipers, and lesion volume was calculated. Ears were digested enzymatically for 90 min at 37°C in 0.25 mg/mL Liberase (Roche) with 10 μg/mL DNase I (Sigma) in RPMI 1640 (Gibco). Tissue parasite burden was determined using limiting dilution assays (45).

### Single-cell RNA sequencing sample preparation

The single-cell RNA sequencing (scRNA-Seq) samples were prepared, and data were acquired as a part of a previous study (38). In short, the ACRI Genomics and Bioinformatics Core prepared NGS libraries from fresh single-cell suspensions using the 10× Genomics NextGEM 3′ assay for sequencing on the NextSeq 500 platform using Illumina SBS reagents. Trypan Blue exclusion determined cell quantity and viability. Library quality was evaluated with the Advanced Analytical Fragment Analyzer (Agilent) and Qubit (Life Technologies) instruments.

### scRNA-Seq data analysis

Data analysis was performed as a part of a previous study (38). Briefly, the UAMS Genomics Core generated demultiplexed fastq files, which were analyzed using 10X Genomics Cell Ranger alignment and gene counting software, a self-contained scRNA-Seq pipeline developed by 10X Genomics. The reads were aligned to the mm10 reference transcriptomes using STAR, and transcript counts were generated (40, 46). The *Seurat* R package processed the raw counts generated by *cellranger count* (47, 48). Potential doublets, low-quality cells, and cells with a high percentage of mitochondrial genes were filtered out. Cells that have unique feature counts > 75th percentile plus 1.5 times the interquartile range (IQR) or <25th percentile minus 1.5 times the IQR were filtered. Similarly, cells with mitochondrial counts falling outside the same range for mitochondrial gene percentage were filtered. After filtering, all eight sequencing runs were merged. The counts were normalized using the LogNormalize method, which log transforms the results (38). Subsequently, the 2,000 highest variable features were selected. The data were scaled, and principal component analysis (PCA) was performed. A JackStraw procedure was implemented to determine the significant PCA components that have a strong enrichment of low *P*-value features.

A graph-based clustering strategy was used to embed cells in a graph structure (49). Seurat visualized the results in t-distributed stochastic neighbor embedding and uniform manifold approximation and projection (UMAP) plots (50). Seurat FindNeighbors and FindClusters functions were optimized to label clusters. Seurat FindAllMarkers function finds markers that identify clusters by differential expression, defining positive markers of a single cluster compared to all other cells and comparing those to known markers of expected cell types from previous single-cell transcriptome studies. Cell type identifications were determined by manually reviewing these results, and some clusters were combined if their expression was found to be similar. From here for this work, we specifically provide feature maps showing transcript expression of Gbp2, Gbp3, Gbp5, Gbp7, and Gbp9 among all clusters, particularly in macrophages and monocytes.

### Flow cytometry

Flow cytometric analysis was conducted *in vivo* on ear tissue. For *in vivo* flow cytometric analysis, ear tissue was enzymatically digested and processed for flow cytometric analysis of dermal cells from the ears. To determine cellular viability, cells were incubated with a Zombie Aqua viability dye (Biolegend). Fc receptors were blocked with 2.4G2 anti-mouse CD16/CD32 (Invitrogen or BioXCell) and 0.2% rat IgG (BioXCell). Cells were surface stained with antibodies against CD45 BV650 (BD Horizon) or AF700 (eBioscience), CD11b BV750 (Biolegend), or BV605 (Biolegend), CD64 BV421 (Biolegend) or BV711 (Biolegend), Ly6C PercpCy5.5 (Invitrogen), and Ly6G AF700 (Biolegend). Intracellular stain was performed using the Foxp3 Transcription Factor Staining Buffer Kit (Invitrogen). Intracellular molecules and cytokines were stained with antibodies against Arg-1 PE (Invitrogen) or iNOS APC (Biolegend). Cell events were acquired on a Cytek Northern Lights (Cytek) and analyzed using FlowJo (Tree Star).

### Statistics

All data were analyzed for statistical significance using GraphPad Prism 9. Statistical significance was determined using a two-tailed Student’s unpaired *t*-test or a two-way ANOVA with a Tukey’s multiple comparison test. Outliers were identified by Grubb’s outlier test and removed.

## RESULTS

Gbps orchestrate the host defense to many intracellular pathogens. To determine if Gbps are involved in the host defense against *Leishmania* parasites, we analyzed the expression of Gbps during *in vivo L. major* infection. Briefly, C57BL/6 mice were intradermally infected with *L. major* parasites in the ear, and at 4 weeks post-infection (wpi), RNA sequencing was conducted on whole lesions from mice infected with *L. major* and compared to naive controls (Fig. 1A). Multiple Gbps were upregulated with *L. major* infection, including *Gbp2*, *Gbp3*, *Gbp4*, *Gbp5*, *Gbp7*, *Gbp8*, and *Gbp9* (Fig. 1A).

To validate our RNA-seq findings and identify the most highly upregulated Gbps during *L. major* infection, we performed real-time PCR to quantify Gbp expression relative to naive samples. By utilizing log₂ fold change as a comparative metric, we prioritized candidates for further investigation. Gbp2, Gbp5, and Gbp9 were most upregulated with *L. major* infection compared to naive samples (Fig. 1B). After confirming that Gbp expression was associated with *L. major* infection, we next determined whether this upregulation is specific to *L. major* or also occurs in response to infections with other *Leishmania* species. Mice were infected with either *L. major*, *L. mexicana*, or *L. amazonensis* parasites intradermally in the ear. Following the establishment of infection and lesion formation, ear tissue was collected and prepared for RT-PCR (Fig. 1C). Interestingly, *Gbp2* and *Gbp9* were also elevated following infection with both *L. mexicana* and *L. amazonensis* (Fig. 1C). The expression of *Gbp3* and *Gbp7* increased following infection with *L. mexicana* (Fig. 1C). These data suggest that multiple Gbps are elevated *in vivo* after infection with different *Leishmania* species, and that Gbp expression is a hallmark of CL, although different Gbps may be elevated depending on the species of infecting parasite.

After confirming that Gbp expression is elevated during *L. major* infection, we next analyzed the cell types expressing Gbps during *in vivo L. major* infection. We hypothesized that Gbps are expressed in myeloid-derived cells during *L. major* infection, as these are the cells responsible for harboring and killing parasites. To determine which cell types express Gbps during infection, mice were infected with *L. major* parasites, and at 4 wpi, tissue was taken for single-cell RNA sequencing from naive skin and CL lesions as a part of a previous study (38). The scRNA-Seq data revealed 35 distinct cell populations in the lesion present after *L. major* infection (Fig. 2A). We analyzed the expression of *Gbp2*, *Gbp3*, *Gbp5*, *Gbp7*, and *Gbp9,* and the findings are consistent between each Gbp. In the naive uninfected skin tissue, resident macrophages were the main cell type expressing Gbps (Fig. 2A and B). Additionally, we analyzed violin plots of the Gbps most highly upregulated during *L. major* infection compared to naive tissue. Interestingly, during *L. major* infection, macrophages, particularly monocyte-derived macrophages and resident macrophages, were the main cell types expressing Gbps (Fig. 2B and C). Monocyte-derived macrophages originate from inflammatory monocytes recruited to the infection site to replenish resident macrophages. We found that iMonos also exhibited elevated Gbp expression with *L. major* infection, but to a lesser extent than monocyte-derived macrophages and resident macrophages. This is relevant as both resident macrophages, monocyte-derived macrophages, and iMonos become infected with *L. major* and are responsible for either serving as a permissive niche facilitating infection or as a resistant host cell orchestrating parasite killing. Additionally, fibroblasts, T cells, and neutrophils expressed Gbps during *L. major* infection. Altogether, these data highlight the preferential, yet unrestricted expression of Gbps in myeloid cells.

**Fig 3:**
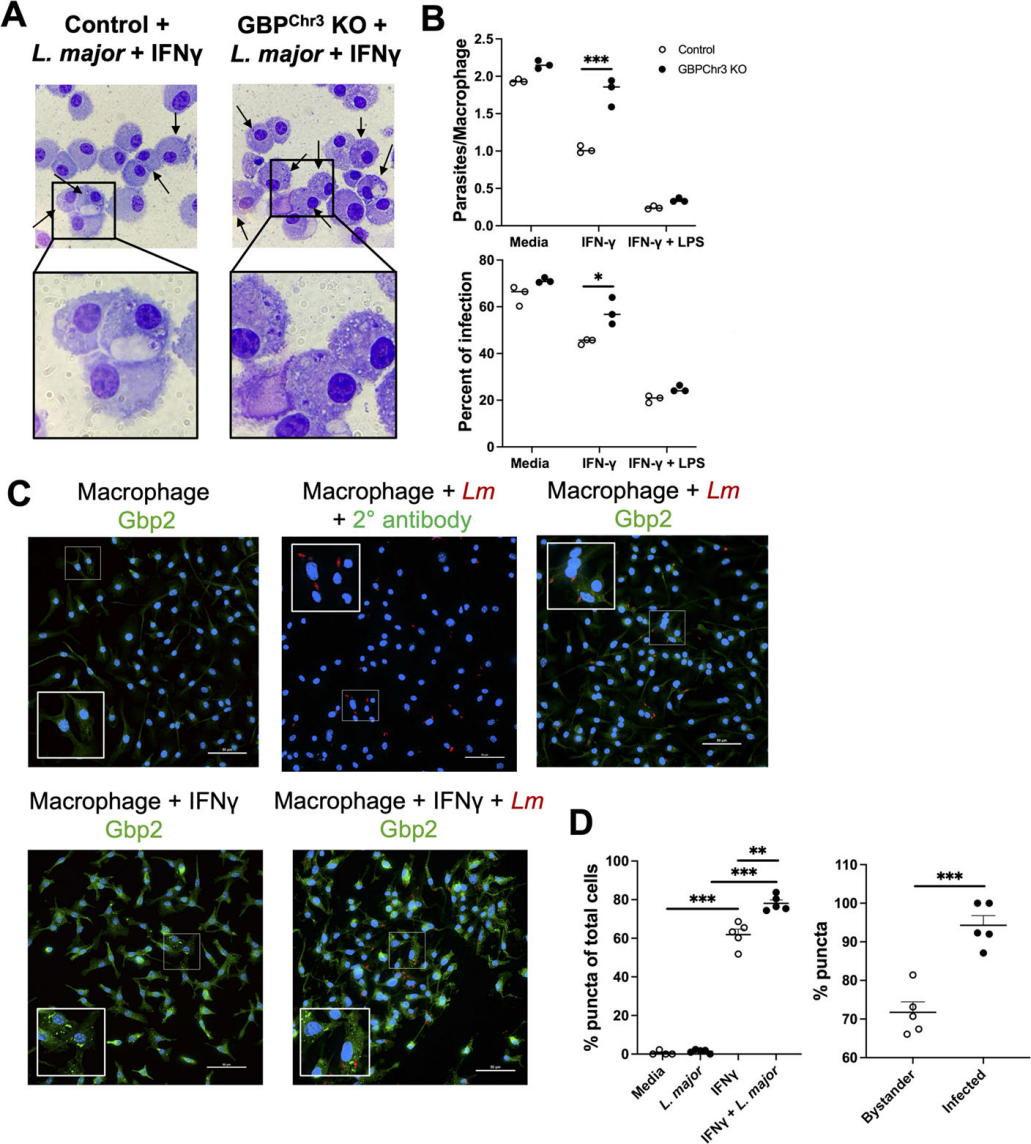
Macrophage Gbps control *L. major* parasites independent of localization to the parasite membrane. BMDMs from control mice or Gbp^Chr3^ KO mice were infected at an MOI of 1:10 for 2 h before extracellular parasites were washed away. Infected cells were then cultured in media or different supplemented medium conditions for either 72 or 48 hpi before cells were prepped for cytospin analysis or immunofluorescence microscopy, respectively. (**A**) Representative images of control or Gbp-deficient macrophages infected with *L. major* and treated with IFN-γ for 72 h to assess parasite survival. (**B**) Quantification of panel **A** showing parasites per macrophages and percentage of infection after infection alone, infection and treatment with IFN-γ, or infection and treatment with LPS + IFN-γ after 72 hpi. (**C**) Following infection with DsRed *L. major* infection, cells were cultured in supplemented media for 48 h to assess localization to *L. major*. At 48 hpi, cells were fixed and stained with an antibody against Gbp2 for immunofluorescence microscopy. Gbp2 expression is shown in green, DsRed^+^ *L. major* is in red, and DAPI is in blue. Representative images of macrophages cultured in media, infected with *L. major* and only stained with a secondary antibody, infected with *L. major* and stained with an antibody against Gbp2, IFN-γ-treated macrophages, or IFN-γ-treated macrophages infected with *L. major*. (**D**) Quantification of the percentage of puncta containing cells as a percentage of total cells in panel **C,** and quantification of bystander versus infected cells containing puncta in panel **C**. Data are representative of two or three independent experiments. Significance was determined using a two-way ANOVA paired with a Tukey’s multiple comparison test, where *P < 0.05, **P < 0.01, and ***P < 0.001.

Next, to investigate whether Gbps play a role in host defense during *L. major* infection, we assessed parasite burdens using bone marrow-derived macrophages (BMDMs), given that macrophages are the main cell type harboring and killing parasites during *Leishmania* infection. Additionally, Gbps were most highly upregulated in the macrophage population during *L. major* infection (Fig. 2A through C). Specifically, macrophages were derived from control C57BL/6 mice or Gbp^Chr3^ KO mice, which are deficient in all the Gbps contained on chromosome 3, including Gbp1, Gbp2, Gbp3, Gbp5, and Gbp7, as described previously (36). Gbps often work in concert with one another, and the deletion of only one or two Gbps may lead to compensation by other Gbps. Deletion of Gbps contained on chromosome 3 has shown efficacy in studying the role of Gbps in health and disease (51–54). These Gbps are more often associated with roles in the immune response (20, 24, 25, 51). To analyze parasite burdens in macrophages with and without Gbps, we differentiated macrophages from bone marrow cells from either control or Gbp^Chr3^ KO mice. After differentiation, BMDMs were infected with *L. major* for 2 hpi before extracellular parasites were washed away. Infection for 2 hpi was followed by treatment with IFN-γ, or IFN-γ and LPS. Cytospin was used to calculate parasite burdens (Fig. 3A and B). We observed no significant differences between parasites per macrophage or the percent infectivity of control or Gbp^Chr3^ KO macrophages during infection with *L. major* after 72 h of culture in media (Fig. 3B). However, upon treatment with IFN-γ, Gbp^Chr3^ KO macrophages harbored significantly more parasites per macrophage as calculated by parasites per total macrophages (both infected and uninfected) (Fig. 3A and B). Additionally, the percentage of infected macrophages, calculated by comparing infected and uninfected macrophages, was higher for Gbp^Chr3^ KO macrophages compared to controls that were infected and treated with IFN-γ (Fig. 3A and B). We also analyzed parasite burdens in control or Gbp^Chr3^ KO macrophages treated with LPS and IFN-γ, which is used as a positive control for parasite killing due to high levels of NO produced in this condition. There were no differences in parasite burden in control or Gbp^Chr3^ KO macrophages infected and treated with LPS/IFN-γ, suggesting the defect in parasite control can be overcome depending on the external stimuli (Fig. 3B). These data suggest that upon stimulation with IFN-γ, Gbps play a role in parasite control, and during Gbp deficiency, macrophages are less equipped to combat the infection.

Gbp localization to vacuole membranes is intimately tied to function and pathogen control (55, 56). Therefore, we investigated whether Gbps localizes to *L. major* to determine if Gbp function during *L. major* infection is tied to cellular localization. We chose to analyze Gbp2 protein based on our transcriptomic data, where Gbp2 is the most highly elevated during infection with *L. major* compared to the other Gbps (Fig. 1). To analyze Gbp2 localization during *L. major* infection, BMDMs were cultured in media and either left uninfected, infected with *L. major* for 2 h, treated with IFN-γ, or infected for 2 h, followed by treatment with IFN-γ. At 48 hpi, samples were stained with an antibody against Gbp2. First, to determine the expression of Gbp2 during homeostasis, Gbp2 expression was analyzed in macrophages cultured in media alone (Fig. 3C). We detected a diffuse signal of Gbp2 within uninfected untreated cells (Fig. 3C). Importantly, the signal detected in untreated macrophages was not due to the background of the secondary antibody. When untreated macrophages were stained with the secondary antibody alone, there was no diffuse signal detected within the cells, suggesting that during homeostasis, Gbp2 is expressed ubiquitously throughout the cell (Fig. 3C). During infection of untreated macrophages, we observed similar Gbp2 expression compared to uninfected BMDMs cultured in media alone (Fig. 3C). However, upon treatment with IFN-γ, Gbp2 was highly expressed and detected throughout the cell, manifesting mostly as puncta (Fig. 3C). The Gbp2 localization during treatment with IFN-γ in the absence of parasites was spread out throughout the cell (Fig. 3C). Interestingly, upon *L. major* infection and IFN-γ treatment, Gbp2 was similarly localized throughout the cell compared to treatment with IFN-γ alone (Fig. 3C). Infected and uninfected bystander macrophages stimulated with IFN-γ possessed punctate Gbp2 throughout the cell (Fig. 3C). Importantly, we did not detect Gbp2 signal localized to the parasite membrane, although many Gbp puncta were detected in close association with the parasite signal. These data suggest that macrophage activation leads to increased Gbp2 protein; however, although Gbps contribute to the control of *L. major* parasites, this activity is not achieved through Gbp2 localization to the intracellular parasite.

We next measured the percentage of cells with Gbp2 puncta within each condition. We only detected Gbp puncta in infected, bystander, and uninfected cells that were treated with IFN-γ and not in macrophages with no pro-inflammatory stimulus (Fig. 3C). Interestingly, we found that BMDMs treated with both IFN-γ and *L. major* possessed more puncta-positive cells than those treated with IFN-γ alone (Fig. 3D). Next, we investigated which cells possess puncta, specifically infected cells or in nearby uninfected bystander cells, during treatment with IFN-γ and infection with *L. major*. Interestingly, many bystander cells contained Gbp2 puncta (Fig. 3D). However, the percentage of puncta-positive cells was significantly higher among infected cells compared to bystander uninfected cells (Fig. 3D). These data together suggest that Gbp2 puncta are significantly increased in infected macrophages compared to bystander cells.

**Fig 4:**
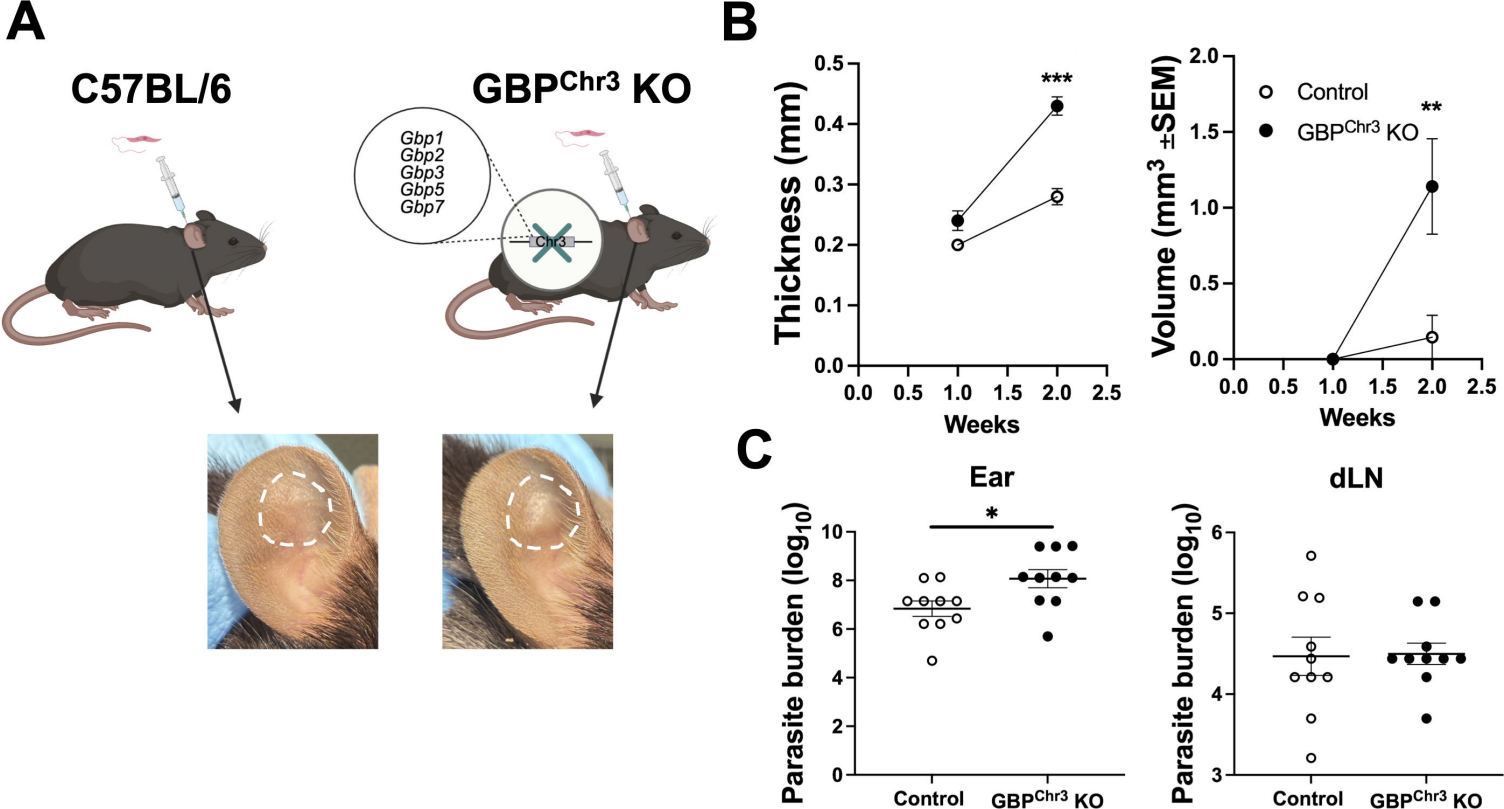
Gbps participate in the control of parasites and disease severity during *L. major* infection. C57BL/6 control or Gbp^Chr3^ KO mice were infected intradermally with 2 × 10^6^ *L. major* parasites. (**A**) Diagram showing infection of control or Gbp^Chr3^ KO mice and representative lesions from each mouse strain at 2 wpi. (**B**) Lesions were monitored at 1 and 2 wpi by measuring ear thickness and lesion volume with electronic calipers. (**C**) The local ear and systemic dLN parasite burdens were quantified using a limiting dilution assay at 2 wpi. Data are pooled from three experiments with five mice per group per experiment. Significance was determined using a *t*-test, where *P < 0.05, **P < 0.01, and ***P < 0.001.

Because Gbp expression is increased with infection, and IFN-γ-treated Gbp^Chr3^ KO macrophages harbor more parasites compared to control macrophages, we next investigated if Gbps play a role in parasite control or disease pathology during *in vivo L. major* infection. To analyze Gbps during *in vivo L. major* infection, we utilized the previously mentioned mouse strain deficient in the Gbps located on chromosome 3. To initially determine if Gbps are important to host defense during *L. major* infection, control or Gbp^Chr3^ KO mice were intradermally infected with *L. major* parasites, and lesion development was monitored weekly (Fig. 4A). Interestingly, Gbp^Chr3^ KO mice exhibited significantly larger lesions at 2 wpi compared to control infected mice, where both the thickness and volume are significantly larger than controls (Fig. 4A and B). Despite early differences in lesion progression between control and Gbp^Chr3^ KO mice, Gbp^Chr3^ KO mice ultimately heal their lesions similarly to control mice (Fig. S1A and B). To understand the role of Gbps during the first 2 weeks of infection with *L. major*, we analyzed local and systemic parasite burdens at 2 wpi. Gbp^Chr3^ KO mice possess higher local parasite burdens in the skin compared to control mice at 2 wpi (Fig. 4C). Systemic parasite burdens in the draining lymph node (dLN) were unchanged compared to control mice at 2 wpi (Fig. 4C). These data suggest that Gbps play a role in disease control during *L. major* infection, and in the absence of Gbps, parasite burden is elevated, resulting in worse disease severity at 2 wpi (38).

Because we previously determined that macrophages are the main cell type expressing Gbps during *L. major* infection, we investigated macrophages from WT and Gbp^Chr3^ KO mice during *L. major* infection. Given that macrophages most highly express Gbps during *L. major* infection, we hypothesized that the macrophage population is responsible for the difference in parasite burden in Gbp^Chr3^ KO mice compared to controls. Additionally, since we did not observe Gbp localization to *L. major in vitro*, we hypothesize that the difference in parasite burden between control and Gbp^Chr3^ KO mice was attributed to an alternative mechanism of control by Gbps. To further understand the role of Gbps in parasite control, we analyzed the cellular phenotype of macrophages during *L. major* infection in mice with and without Gbps. Specifically, we employed flow cytometry to assess macrophage iNOS and arginase-1 (Arg-1) levels. We chose to analyze these two molecules because iNOS expression is generally associated with NO production and parasite control, while Arg-1 is associated with susceptible hosts and parasite survival in leishmaniasis. Specifically, Arg-1 leads to the synthesis of polyamines, which directly contribute to parasite replication (57). So, iNOS and Arg-1 positivity is used as a surrogate to probe between M1 versus M2 macrophages within tissues. To assess macrophage phenotype, mice were infected intradermally with *L. major* parasites, and at 2 wpi, ear tissue was subjected to flow cytometry.

**Fig 5:**
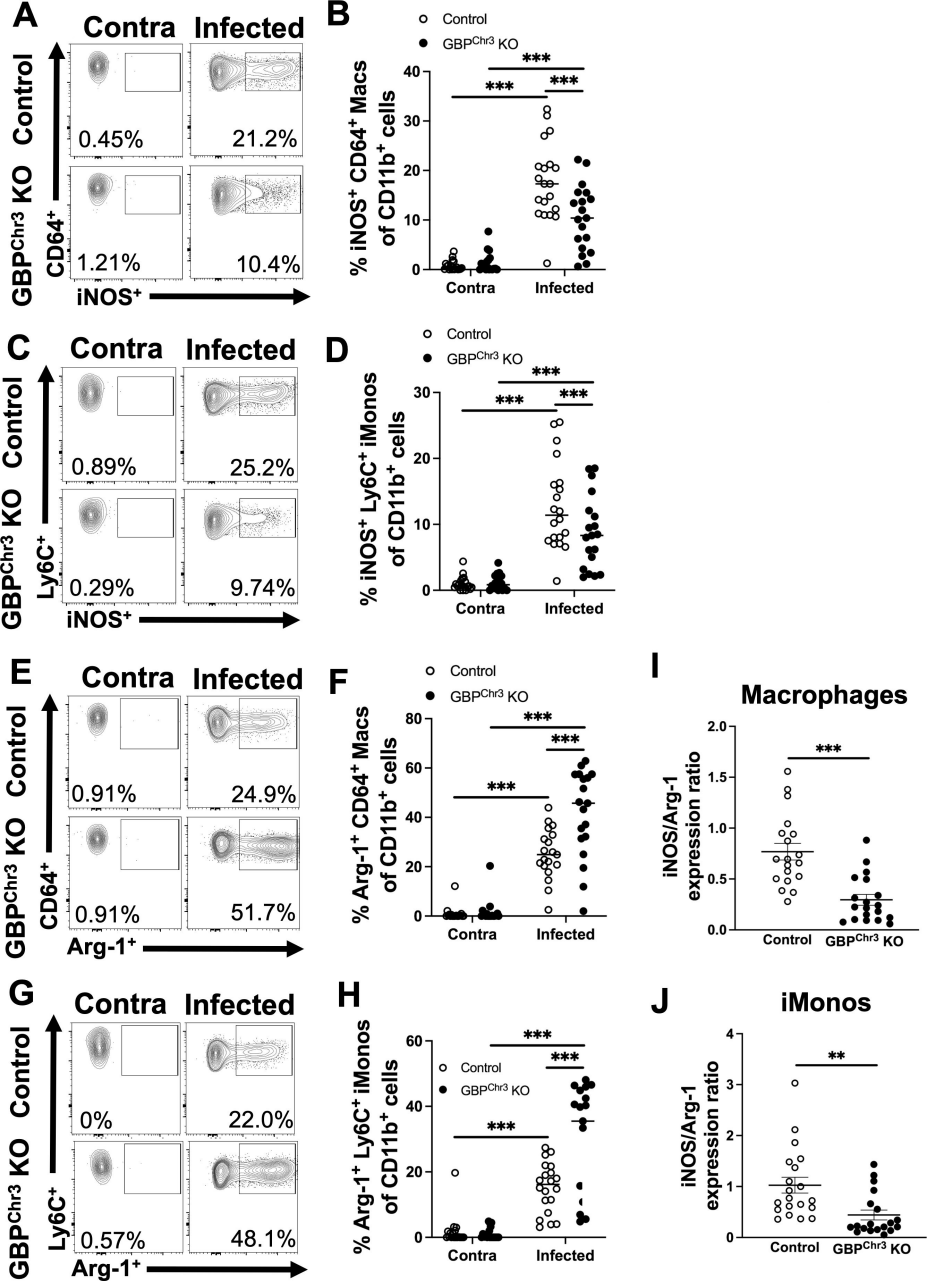
Gbp deficiency results in reduced iNOS^+^ myeloid cells and increased Arg-1^+^ myeloid cells. Mice were infected with *L. major* parasites, and at 2 wpi, flow cytometric analysis was performed on ear tissue from control or Gbp^Chr3^ KO mice. (**A**) Representative flow plots of iNOS^+^ macrophages are shown, gated on live, single, CD45^+^, CD11b^+^, CD64^+^, and Ly6G^−^ cells. (**B**) Quantification of panel **A** shows the percentage and number of iNOS^+^ macrophages in the infected and contralateral ear of control or Gbp^Chr3^ KO mice. (**C**) Representative flow plots of iNOS^+^ iMonos are shown, gated on live, single, CD45^+^, CD11b^+^, Ly6C^+^, and Ly6G^−^ cells. (**D**) Quantification of panel **C** shows the percentage and number of iNOS^+^ iMonos in the infected and contralateral ear of control or Gbp^Chr3^ KO mice. (**E**) Representative flow plots of Arg-1^+^ macrophages are shown. (**F**) Quantification of panel **E** shows the percentage and number of Arg-1^+^ macs. (**G**) Representative flow plots of Arg-1^+^ iMonos are shown. (**H**) Quantification of panel **G** shows percentage and number of Arg-1^+^ iMonos. (**I**) Expression ratio of iNOS^+^ macrophages to Arg-1^+^ macrophages. (**J**) Expression ratio of iNOS^+^ iMonos to Arg-1^+^ iMonos. Data are pooled from three experiments with 5–10 mice per group per experiment. Data are shown as the mean. Significance was determined using a two-way ANOVA paired with a Tukey’s multiple comparison test, where *P < 0.05, **P < 0.01, and ***P < 0.001.

We assessed the percentage and total number of iNOS^+^ macrophages and iMonos. We identified a significant decrease in the percentage of iNOS^+^ macrophages in lesions in Gbp^Chr3^ KO mice compared to control mice (Fig. 5A and B). We observed a similar effect for iMonos as well, where Gbp^Chr3^ KO mice exhibited a lower percentage of iNOS^+^ iMonos compared to controls (Fig. 5C and D). Additionally, not only were the iNOS^+^ populations of macrophages and iMonos reduced, but we also observed a significant increase in the percentage of macrophages and iMonos that displayed Arg-1 positivity (Fig. 5E through H). Furthermore, the expression ratio of iNOS^+^ cells to Arg-1^+^ cells was significantly decreased for both macrophages and iMonos (Fig. 5I and J). These data suggest that myeloid lineage cells within Gbp^Chr3^ KO are more characteristic of M2-like cells, which are associated with parasite survival and disease progression. Together, these data suggest that immune cells from Gbp^Chr3^ KO mice possess an enhanced Arg-1 M2 program. Importantly, during Gbp deficiency and *L. major* infection, the lesional macrophages and iMonos display a parasite-friendly environment, consistent with higher parasite burdens in the Gbp^Chr3^ KO mice compared to control mice.

## DISCUSSION

Gbps are involved in the host response to a wide variety of intracellular pathogens (19). In this study, we demonstrated for the first time that Gbps play a role in host defense and parasite control during an *in vivo* experimental model of *Leishmania* infection. Specifically, we found that various Gbps were upregulated following infection with *L. major*, *L. mexicana*, and *L. amazonensis*. Previous work found that infection with *L. major* in mice of different genetic backgrounds is associated with the expression of Gbp2 and Gbp5 (32). However, this prior work did not establish a direct role for Gbps in parasite control. Moreover, it was previously determined that during *L. major* infection, Gbp2 is highly expressed in the skin tissue of semi-resistant mouse models within infected cells (32). In contrast, in the highly susceptible BALB/c mouse strain, Gbp expression is decreased, and less-infected cells express Gbps (32). This suggests that Gbps may influence disease outcome, with more favorable disease outcomes occurring when Gbps are highly expressed in cells infected with *Leishmania* parasites. Whether this holds true for infections with *Leishmania* species that cause more severe disease outcomes, such as *L. amazonensis* and *L. mexicana*, remains to be determined and is an active area of investigation in the lab. 

In this work, we define that the loss of Gbps during infection with *L. major* parasites increases parasite burdens compared to controls *in vivo*. We speculate that this is due to an increase in the percentage of Arg-1^+^ CD64^+^ macrophages and a decrease in iNOS^+^ CD64^+^ macrophages, reflecting a phenotypic shift in the macrophage population. The iNOS/Arg-1 ratio is a key indicator of whether the environment is permissive or resistant to parasites (58, 59). Specifically, cases of *Leishmania* infection characterized by a robust M2 macrophage response are associated with worse disease outcomes and uncontrolled parasite replication (58, 60, 61). For example, during *L. major* infection in both C57BL/6 and BALB/c mice, an Arg-1^+^ population is present (62). However, in susceptible BALB/c mice, arginase expression begins around lesion formation and progressively increases until the experimental endpoint (62). In contrast, during infection in resistant C57BL/6 mice, arginase expression peaks at 3 wpi, when lesions are present, and then gradually declines as the lesion resolves (62). While we speculate that macrophages are leading to the increased parasite burdens in the absence of Gbps, stromal cells like fibroblasts also express Gbps during *L. major* infection, and the role of these non-hematopoietic cell types and other immune cells has not been ruled out and remains an exciting area of exploration. 

However, a caveat to our study is that we did not uncouple whether IFN-γ suppresses parasite growth or leads to killing in wild-type macrophages; we plan to investigate the mechanism by which IFN-γ decreases parasite numbers and how that process is altered in Gbp^Chr3^ KO macrophages in the future. Notably, iNOS positivity is not limited to infected cells (63). In fact, recruited monocytes rapidly induce iNOS, with the percentage of iNOS^+^ cells exceeding the percentage of infected cells (63). Importantly, the rapid induction of iNOS was linked to both TNF-α and IFN-γ production (63). Whether these factors are intact in the skin during infection in Gbp-deficient mice remains to be determined. Furthermore, we detected elevated levels of activated TNF-α-positive CD4^+^- and CD8^+^-activated T cells in the dLNs, suggesting that the T cell compartment may be compensating for the reduction in iNOS expression in myeloid cells (Fig. S2A through D). Additionally, splenocytes derived from either infected control or Gbp^Chr3^ KO mice, cultured in either medium with *L. major* antigen or with TCR stimuli, exhibit enhanced TNF-α production (Fig. S2E). Taken together, these data suggest that during infection with *L. major*, Gbps orchestrate the NO gradient, which is crucial for subsequent parasite control.

Mice deficient in iNOS and infected with *L. major* display increased lesion sizes compared to control mice starting at 4 wpi and continuing through 10 wpi (64). This suggests that, although Gbp^Chr3^ KO mice exhibit a defect in the iNOS^+^ cellular compartment, their phenotype does not entirely match that of iNOS-deficient mice. We observed significant differences in lesion sizes between Gbp^Chr3^ KO mice and controls at 2 and 3 wpi. However, the difference in lesion size between groups disappeared by 4 wpi, with Gbp^Chr3^ KO mice ultimately healing their lesions in a manner similar to control mice (Fig. S1). This indicates that, despite an initial defect in parasite control during Gbp^Chr3^ deficiency, the host can overcome differences in parasite burdens, suggesting compensation by another mechanism after 4 wpi. However, whether Gbp^Chr3^ deficiency affects disease progression in more severe cases of CL, such as infection with *L. amazonensis* or *L. mexicana*, remains unknown and is an ongoing area of research in our lab. 

Previous work showed that during *T. gondii* infection, which stimulates Gbp recruitment to the PV, Gbp2^+^ PVs are highly enriched for iNOS activity (55). In this context, Gbp2 and iNOS appear to work in concert, with inhibition of iNOS leading to the shedding of Gbp2 from the PV and increased parasite replication (55). Ultimately, iNOS was found to be necessary for the control of *T. gondii*, suggesting that Gbps on Chr3 play a key role in orchestrating optimal iNOS-mediated parasite clearance (55). In our study, we determined that Gbp2 does not localize to *L. major* during *in vitro* infection (Fig. 2). Specifically, we found that following IFN-γ treatment, Gbps are expressed throughout the cell, and upon infection with *L. major*, Gbp2 localization does not change, and Gbp2 does not migrate to *L. major*. Consistent with our findings, previous research with *L. donovani* infection found that Gbps do not localize to the PV in non-phagocytic cells (31). During bone marrow-derived monocyte/DC infection with *T. gondii*, Gbp2 forms a tight ring around the PV containing *T. gondii,* and iNOS is closely associated with Gbp2^+^ PVs. However, investigation with hGBPs during *T. gondii* infection indicates both distal and proximal roles for Gbps, suggesting other Gbps during *L. major* infection may be recruited to the vacuole (65). Regardless, our findings indicate that while Gbps may coordinate iNOS expression in our model, this coordination is not directly linked to Gbp2 localization like *T. gondii* infection.

### Conclusion

Ultimately, this work uncovers a critical host macrophage intrinsic mechanism in the control of *L. major* parasites. Current anti-leishmanial therapies, such as miltefosine, promote the production of IFN-γ, which subsequently induces ROS and NO production (66). However, the extent to which the therapeutic effects of these treatments are mediated through the activation of Gbps remains unclear. Future studies should focus on characterizing Gbp responses during anti-leishmanial treatment to elucidate their potential role in enhancing parasite control. Targeting Gbps may hold therapeutic promise by promoting the expansion of iNOS^+^ macrophages, thereby improving the host’s ability to combat *L. major* and potentially other *Leishmania* species.

## Data Availability

The raw data from our bulk RNA-Seq analysis were deposited in Gene Expression Omnibus (GEO accession number GSE185253).

## SUPPLEMENTAL MATERIAL

Supplemental Figures mbio.02825-25-s0001.pdf Fig. S1 and S2.

ASM does not own the copyrights to Supplemental Material that may be linked to, or accessed through, an article. The authors have granted ASM a non-exclusive, world-wide license to publish the Supplemental Material files. Please contact the corresponding author directly for reuse.
