# Supplementary material for: Guanylate-binding proteins balance iNOS/Arg-1 in myeloid cells during L. major infection and promote host defense to infection
Source: mBio. 2026 Mar 10;17(4):e02825-25. doi: 10.1128/mbio.02825-25 (PMC13059736; doi:10.1128/mbio.02825-25)
Supplement: Supplemental Figures — Fig. S1 and S2. [file mbio.02825-25-s0001.pdf]

Supplemental Figures

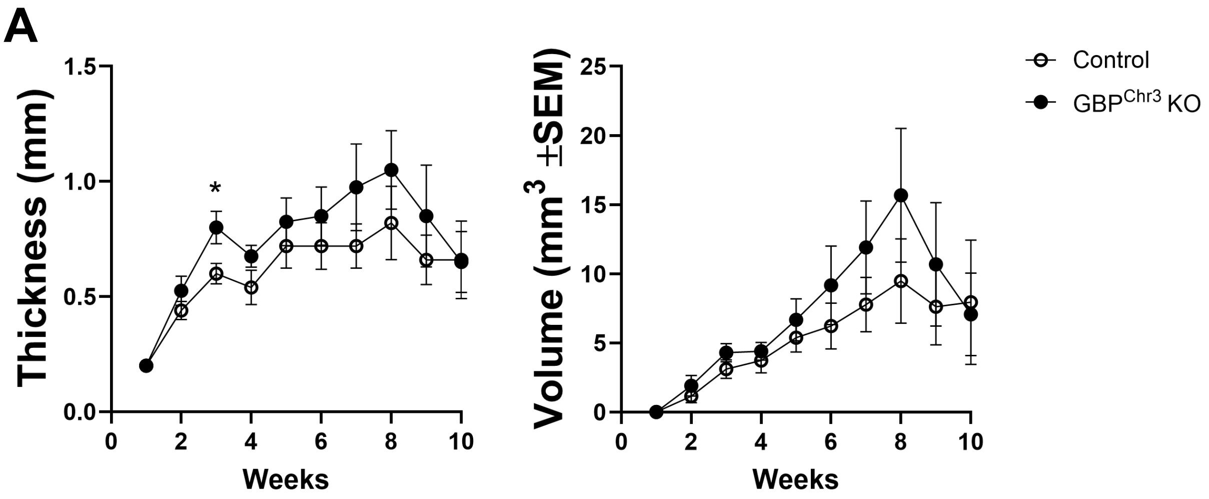

Figure S1: Lesions developed in GBP^{Chr3} KO mice ultimately resolve, similar to control mice despite an initial difference in disease severity. C57BL/6 control or GBP^{Chr3} KO mice were infected intradermally with $5x10^6$ *L. major* parasites. (A) Lesions were monitored weekly by measuring ear thickness and lesion volume with electronic calipers. Data is representative of 2 experiments where n=5 or n=10 per group per experiment. Significance was determined using a t-test where *p<0.05.

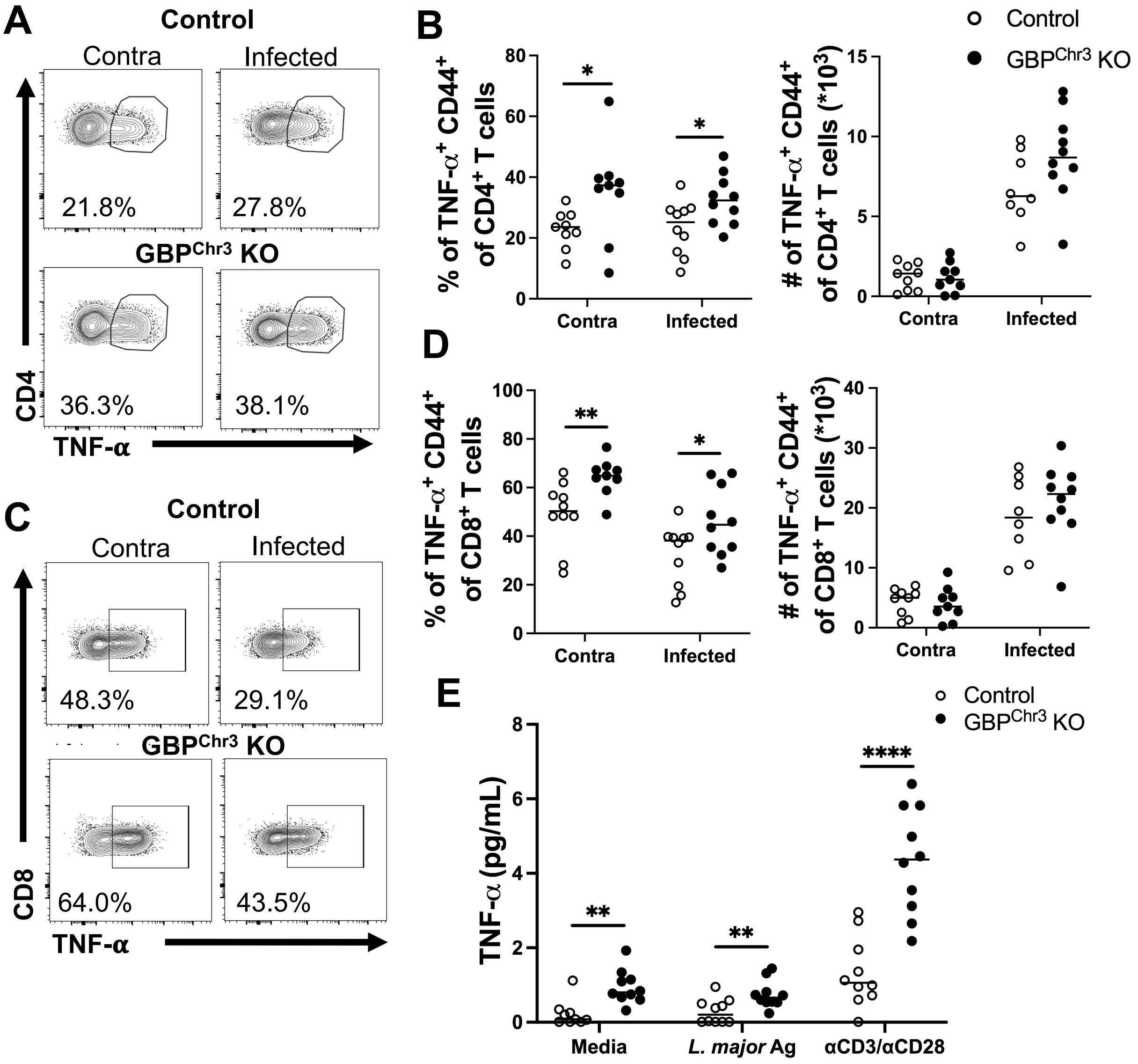

Figure S2: CD4⁺ and CD8⁺ T cells exhibit enhanced inflammatory profiles in GBP^{Chr3} KO mice compared to control mice. Mice were infected with *L. major* parasites and at 2 wpi flow cytometric analysis was performed on dLN tissue from control or GBP^{Chr3} KO mice. (A) Representative flow plots of TNF-α^{+} CD44^{+} CD4^{+} T cells are shown. Gated on live, single, CD45^{+}, CD3^{+} cells. (B) Quantification of (A) shows percentage and number

of TNF-α^+ activated $CD4^+$ T cells in the dLN of infected or contralateral of control or GBP^{Chr3} KO mice. (C) Representative flow plots of TNF-α^+ activated $CD8^+$ T cells are shown. Gated on live, single, $CD45^+$, $CD3^+$ cells. (D) Quantification of (C) showing percentage and number of TNF-α^+ $CD8^+$ T cells. (E) Splenocytes from infected control or GBP^{Chr3} KO mice were cultured in media, with *L. major* Ag, or with TCR-activating stimuli anti-CD3/anti-CD28. After 72 hours supernatants were collected and an ELISA quantified TNF-α. Data are representative of 2 independent experiments where n=5 or n=10 mice per group. Data are shown as mean. Significance was determined using a two-way ANOVA paired with a Tukey's multiple comparison test where $^*p<0.05$ $^{**}p<0.01$, $^{***}p<0.001$.
